# 11-Oxygenated C19 Steroids Are the Predominant Androgens in Polycystic Ovary Syndrome

**DOI:** 10.1210/jc.2016-3285

**Published:** 2016-11-30

**Authors:** Michael W. O’Reilly, Punith Kempegowda, Carl Jenkinson, Angela E. Taylor, Jonathan L. Quanson, Karl-Heinz Storbeck, Wiebke Arlt

**Affiliations:** 1 Institute of Metabolism and Systems Research, University of Birmingham, Edgbaston, Birmingham B15 2TT, United Kingdom;; 2 Centre for Endocrinology, Diabetes and Metabolism, Birmingham Health Partners, Edgbaston, Birmingham B15 2TH, United Kingdom;; 3 Department of Biochemistry, Stellenbosch University, Stellenbosch 7600, South Africa; and; 4 National Institute of Health Research (NIHR) Birmingham Liver Biomedical Research Unit, University Hospital Birmingham, NHS Foundation Trust, Birmingham B15 2GW, United Kingdom

## Abstract

**Context:**

Androgen excess is a defining feature of polycystic ovary syndrome (PCOS), but the exact origin of hyperandrogenemia remains a matter of debate. Recent studies have highlighted the importance of the 11-oxygenated C19 steroid pathway to androgen metabolism in humans. In this study, we analyzed the contribution of 11-oxygenated androgens to androgen excess in women with PCOS.

**Methods:**

One hundred fourteen women with PCOS and 49 healthy control subjects underwent measurement of serum androgens by liquid chromatography-tandem mass spectrometry. Twenty-four–hour urinary androgen excretion was analyzed by gas chromatography-mass spectrometry. Fasting plasma insulin and glucose were measured for homeostatic model assessment of insulin resistance. Baseline demographic data, including body mass index, were recorded.

**Results:**

As expected, serum concentrations of the classic androgens testosterone (*P* < 0.001), androstenedione (*P* < 0.001), and dehydroepiandrosterone (*P* < 0.01) were significantly increased in PCOS. Mirroring this, serum 11-oxygenated androgens 11*β*-hydroxyandrostenedione, 11-ketoandrostenedione, 11*β*-hydroxytestosterone, and 11-ketotestosterone were significantly higher in PCOS than in control subjects, as was the urinary 11-oxygenated androgen metabolite 11*β*-hydroxyandrosterone. The proportionate contribution of 11-oxygenated to total serum androgens was significantly higher in patients with PCOS compared with control subjects [53.0% (interquartile range, 48.7 to 60.3) *vs* 44.0% (interquartile range, 32.9 to 54.9); *P* < 0.0001]. Obese (n = 51) and nonobese (n = 63) patients with PCOS had significantly increased 11-oxygenated androgens. Serum 11*β*-hydroxyandrostenedione and 11-ketoandrostenedione correlated significantly with markers of insulin resistance.

**Conclusions:**

We show that 11-oxygenated androgens represent the majority of circulating androgens in women with PCOS, with close correlation to markers of metabolic risk.

## Teaser

We analyzed the androgen metabolome in women with polycystic ovary syndrome by mass spectrometry, revealing that 11-oxygenated androgens represent the majority of circulating androgen excess.

Androgen excess is a defining feature of polycystic ovary syndrome (PCOS), which is one of the most common endocrine disorders in women and is associated with reproductive and metabolic complications (1). The origin of androgen excess in PCOS has not been conclusively determined and may differ with phenotype variability, but it is clear that both ovaries and adrenals contribute to increased circulating androgens in affected women. Previous work has highlighted that women with PCOS show evidence of systemic upregulation of 5*α*-reductase activity (2, 3), which activates testosterone (T) to the most potent androgen, 5*α*-dihydrotestosterone. Recently, it was reported that daughters of women with PCOS show evidence of increased 5*α*-reductase activity during infancy (4) prior to adrenarche- and puberty-related increases in androgen production.

Traditionally, serum T has been used as a biochemical marker for androgen excess in the context of PCOS, but this has been fraught with difficulties, largely due to the low circulating concentrations in women as well as the specificity and sensitivity issues of the assays used. Recently, the T precursor androstenedione (A4) has been shown to be a more sensitive marker of PCOS-related androgen excess and, in combination with T, predictive of metabolic risk (5). These findings were confirmed by another group (6), and the diagnostic value of A4 has been recognized in recent position statements (7).

**Figure 1.**
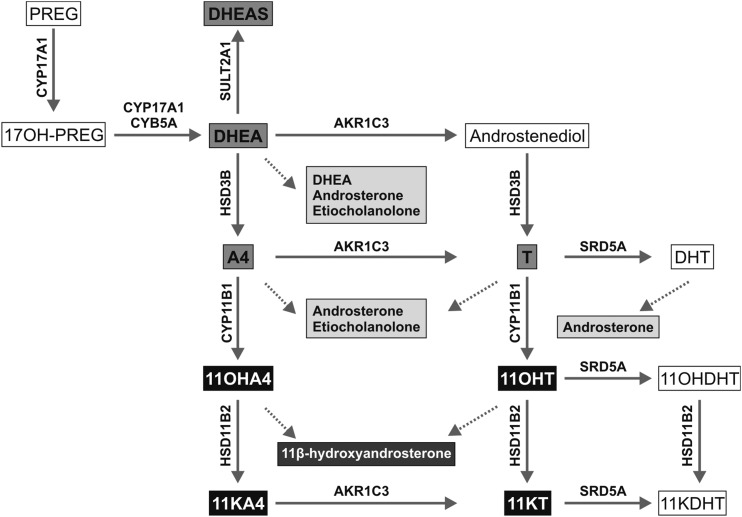
Schematic of androgen synthesis illustrating the classic androgen pathway (gray boxes) and the steroids of the 11-oxygenated androgen pathway (black boxes). Dotted lines relate distinct steroids to their corresponding urinary steroid metabolite. Abbreviations: 11KDHT, 11-ketodihydrotestosterone; 17OH-PREG, 17*α*-hydroxypregnenolone; CYB5A, cytochrome b_5_; CYP11B1, cytochrome P450 11*β*-hydroxylase; CYP17A1, cytochrome P450 17*α*-hydroxylase/17,-20-lyase; DHT, 5*α*-dihydrotestosterone; HSD11B2, 11*β*-hydroxysteroid dehydrogenase type 2; HSD3B, 3*β*-hydroxysteroid dehydrogenase; PREG, pregnenolone; SRD5A, steroid 5*α*-reductase; SULT2A1, sulfotransferase family 2A member 1.

It has been known for decades that the adrenal is capable of converting A4 to 11*β*-hydroxyandrostenedione (11OHA4) (8), catalyzed by the 11*β*-hydroxylase activity of the cytochrome P450 enzyme cytochrome P450 11*β*-hydroxylase, but it was thought to represent an insignificant metabolite. However, recent studies have demonstrated that 11OHA4 is a major product of adrenal steroidogenesis (9) and that its downstream conversion through the 11-oxygenated C19 steroid pathway (Fig. 1) generates 2 steroids, 11-ketotestosterone (11KT) and 11-keto-5*α*-dihydrotestosterone (10), that bind and activate the androgen receptor with affinities and potencies similar to that of T and 5*α*-dihydrotestosterone, respectively (11).

The role of 11-oxygenated C19 steroids in PCOS has not been comprehensively delineated. A number of previous studies have attempted to evaluate the role of 11OHA4 in PCOS, but results have been inconclusive (12–14). To our knowledge, none of these studies examined the role of other 11-oxygenated androgens in global androgen metabolism in women with PCOS. Furthermore, the majority of these early studies relied on radioimmunoassays, now largely surpassed by the advent of modern liquid chromatography-tandem mass spectrometry (LC-MS/MS) assays. Therefore, we have examined the contribution of this poorly characterized androgen pathway to overall androgen excess in a large cohort of women with PCOS by mass spectrometry–based analysis of serum and urinary steroids, in comparison with a healthy female control cohort and in relation to surrogate makers of metabolic risk.

## Materials and Methods

### Subjects and clinical protocol

Women with PCOS aged between 18 and 40 years were recruited from outpatient clinics at University Hospital Birmingham and Birmingham Women’s Hospital. Full ethical approval was obtained from the South Birmingham and Edgbaston Research Ethics Committees (reference numbers LREC5835 and 12/WM/0206). All participants provided written informed consent. PCOS was diagnosed according to the Rotterdam European Society of Human Reproduction and Embryology 2004 criteria, requiring the presence of 2 or more of the following features: oligo/anovulation, clinical signs of hyperandrogenism or biochemical androgen excess, and polycystic ovaries on ultrasound (1). Other potential causes of oligomenorrhea and androgen excess were excluded by history, physical examination, and biochemical assessment. Healthy control subjects were recruited via local advertisement, with the exclusion of PCOS on clinical and biochemical grounds. This was done by obtaining a menstrual history, by direct questioning regarding clinical features of androgen excess, and by biochemical analysis of serum androgens. Individuals with menstrual disturbance and clinical or biochemical hyperandrogenism were excluded. Exclusion criteria for the study were as follows: recent glucocorticoid treatment (within 3 months), pregnancy, age younger than 18 or older than 40 years, recent oral contraceptive use (within 3 months), hyperprolactinemia, thyroid disorders, and frank hyperglycemia.

Study participants attended the National Institutes of Health Research/Wellcome Trust Clinical Research Facility at University Hospital Birmingham after an overnight fast. A precollected 24-hour urine sample for urinary steroid metabolite analysis was provided by each patient on the morning of assessment. Baseline anthropometric data were collected, and blood samples were drawn for fasting glucose and insulin and for measurement of the serum concentrations of classic and 11-oxygenated C19 steroids.

### Serum steroid analysis

All serum steroids were measured by LC-MS/MS. Dehydroepiandrosterone sulfate (DHEAS) was extracted from 20 μL serum by the addition of 20 μL 0.1 mM ZnSO_4_ and 100 μL acetonitrile and quantified in negative mode using a mass spectrometer (Xevo TQ; Waters, Milford, MA) coupled to an ACQUITY UPLC system (Waters) as previously described (5, 15). All other steroids were extracted from 400 μL serum using 2 mL methyl *tert*-butyl ether as previously described (5, 16, 17). Serum T, A4, dehydroepiandrosterone (DHEA), 11OHA4, 11*β*-hydroxytestosterone (11OHT), and 11KT were quantified using a mass spectrometer (Xevo TQ-S; Waters) coupled to an ACQUITY UPLC system (Waters). The serum steroids were separated using a UPLC high-strength silica T3 column (2.1 mm × 50 mm, 1.8 μm) (Waters) and 1% formic acid (A) and 100% methanol (B) as mobile phases. Separation was achieved using a 5-minute linear gradient from 55% A to 75% B at a constant flow rate of 0.6 mL/min and a column temperature of 50°C. All steroids were analyzed in multiple reaction monitoring using the settings reported by Quanson *et al.* (16). Comprehensive validation data for the 11-oxygenated steroids are shown in Supplemental Table 1. Serum 11-ketoandrostenedione (11KA4) was quantified using an ACQUITY UPC^2^ system (Waters) coupled to a mass spectrometer (Xevo TQ-S; Waters) as previously reported (16). Data collection and analysis were performed using MassLynx 4.1 (Waters). Steroids were identified by matching retention times and 2 mass transitions and were quantified by referring to a linear calibration series with appropriate deuterated reference compounds as internal standards.

### Urinary steroid measurement

Urinary steroid metabolites were measured using quantitative gas chromatography-mass spectrometry in selected ion monitoring mode as previously described (18). The steroid metabolites relevant to this study are shown in Table 1 and Figure 1. The production of steroids from the classic androgen pathway was measured by the quantification of the major androgen metabolites androsterone (An) and etiocholanolone (Et). The major 11-oxygenated C19 metabolite 11*β*-hydroxyandrosterone (11*β*-OH-An) was measured to assess the contribution of the 11-oxygenated C19 steroid pathway.

**Table 1.** **Baseline Characteristics and Biochemical Data in the Healthy Control Subjects and the PCOS Cohort, with Additional Comparison of Nonobese and Obese Patients with PCOS**

	**Controls (n = 49)**	**All PCOS (n = 114)**	**Nonobese PCOS (n = 51)**	**Obese PCOS (n = 63)**
Age, y	28 (23–32)	30 (24–36)	29 (24–36)	30 (24–37)
BMI, kg/m^2^	23.7 (21.2–26.1)	31.2 (27.0–36.2)*^a^*	26.0 (23.3–28.0)	35.5 (32.8–38.9)*^a^*^,^*^b^*
HOMA-IR	0.6 (0.4–0.9)	1.7 (0.9–3.6)*^a^*	0.9 (0.5–1.6)	2.7 (1.5–4.9)*^a^*^,^*^b^*
SHBG, nmol/L	58.7 (40.9–81.8)	30.9 (20.8–42.1) *^a^*	36.8 (22.4–57.3)*^a^*	26.7 (18.2–33.9)*^a^*
Serum androgens (nmol/L)				
T	0.3 (0.2–0.5)	0.7 (0.5–1.0)*^a^*	0.7 (0.5–1.0)*^c^*	0.7 (0.5–1.1)*^c^*
A4	5.9 (3.3–9.2)	26.8 (16.9–35.2)*^a^*	24.4 (15.5–35.0)*^a^*	29.2 (17.7–36.0)*^a^*
DHEA	7.1 (4.2–11.8)	14.1 (10.4–18.2)*^c^*	14.7 (10.6–18.8)*^d^*	13.5 (10.4–17.9)
DHEAS (μmol/L)	6.0 (3.4–9.6)	8.1 (5.5–12.2)*^c^*	10.1 (5.6–13.5)*^c^*	7.6 (5.4–11.7)
FAI	0.6 (0.3–0.9)	2.2 (1.4–4.0)*^a^*	1.8 (1.2–3.5)*^d^*	3.0 (1.6–4.3)*^a^*
11OHA4	6.8 (4.9–12.5)	31.7 (16.8–47.8)*^a^*	30.5 (16.1–55.3)*^a^*	34.4 (17.0–46.8)*^a^*
11KA4	2.7 (2.0–3.9)	13.4 (8.5–18.8)*^a^*	13.0 (8.3–17.6)*^a^*	14.2 (8.8–19.8)*^a^*
11OHT	0.2 (0.1–0.3)	0.4 (0.3–0.5)*^c^*	0.4 (0.3–0.5)*^c^*	0.4 (0.3–0.6)*^c^*
11KT	1.5 (1.2–1.8)	2.4 (1.8–3.9)*^c^*	2.4 (1.4–3.4)	2.6 (1.9–4.3)*^c^*
Urinary androgen metabolites (μg/24 h)				
U-An	1231 (856–1814)	2426 (1475–3634)*^c^*	2432 (1451–3719)	2376 (1459–3634)*^d^*
U-Et	1394 (767–1833)	2071 (1305–3005)*^c^*	1991 (1147–2840)	2125 (1425–3077)*^c^*
U-DHEA	388 (145–1209)	536 (185–2009)	590 (129–2171)	461 (198–1832)
U-11*β*-OH-An	353 (171–487)	595 (347–861)*^a^*	595 (438–841)*^c^*	598 (343–899)*^a^*

Abbreviations: FAI, free androgen index; SHBG, sex hormone-binding globulin; U-11*β*-OH-An, urinary 11*β*-hydroxyandrosterone; U-An, urinary androsterone; U-DHEA, urinary dehydroepiandrosterone; U-Et, urinary etiocholanolone.

Data are presented as median and IQR. Statistical comparison was carried out by analysis of variance with *post hoc* Tukey testing. Significance levels are indicated by the footnotes.

^*a*^ *P* < 0.001 as compared with healthy control subjects.

^*b*^ *P* < 0.001 for the comparison nonobese *vs* obese patients with PCOS.

^*c*^ *P* < 0.01.

^*d*^ *P* < 0.05.

### Biochemical analysis

Insulin was measured using a commercially available assay (Mercodia, Uppsala, Sweden) according to the manufacturer’s instructions. Plasma glucose was measured using the 2300 STAT PLUS analyzer (YSI Life Sciences, Yellow Springs, OH). Homeostasis model assessment of insulin resistance (HOMA-IR) was calculated using the formula [fasting glucose (mmol/L)*fasting insulin (mU/L)/22.5].

### Statistical analysis

Data were analyzed using the Statistical Package for the Social Sciences (SPSS), Version 22. Results are presented as median [interquartile range (IQR)] unless otherwise stated. For comparison of single variables, *t* tests (paired or unpaired as appropriate) were used. Nonparametric equivalents where used where data were not normally distributed. One-way analysis of variance with *post hoc* Tukey testing was used for multiple comparisons between different groups. Due to nonnormality of data distribution, Spearman’s Rho was used for correlation testing between continuous variables. Differences were considered statistically significant at *P* < 0.05.

## RESULTS

### Baseline demographics of patients and control subjects

A total of 49 control subjects and 114 women with PCOS were included in the study. After application of the Rotterdam criteria, the prevalence of the 4 Rotterdam-derived phenotypes was as follows: phenotype A (androgen excess, anovulation, and polycystic ovaries), 59.7%; phenotype B (androgen excess, polycystic ovaries), 6.9%; phenotype C (androgen excess, anovulation), 23.6%; phenotype D (anovulation, polycystic ovaries) 9.7%. Control subjects and women with PCOS were matched for age (*P* = 0.18); however, women with PCOS had a significantly higher body mass index (BMI) than age-matched control subjects [median BMI 31.2 kg/m^2^ (IQR, 27.0 to 36.2) *vs* 23.7 kg/m^2^ (IQR, 21.2 to 26.1), respectively; *P* < 0.001] (Table 1). Using a BMI cut-off of 30 kg/m^2^, 51 and 63 women with PCOS were categorized as nonobese and obese, respectively. The BMI in nonobese women with PCOS did not differ from control subjects (*P* = 0.35). HOMA-IR values were significantly higher in obese patients with PCOS than in control subjects (*P* < 0.001).

### Both classic and 11-oxygenated androgens are significantly increased in PCOS

**Figure 2.**
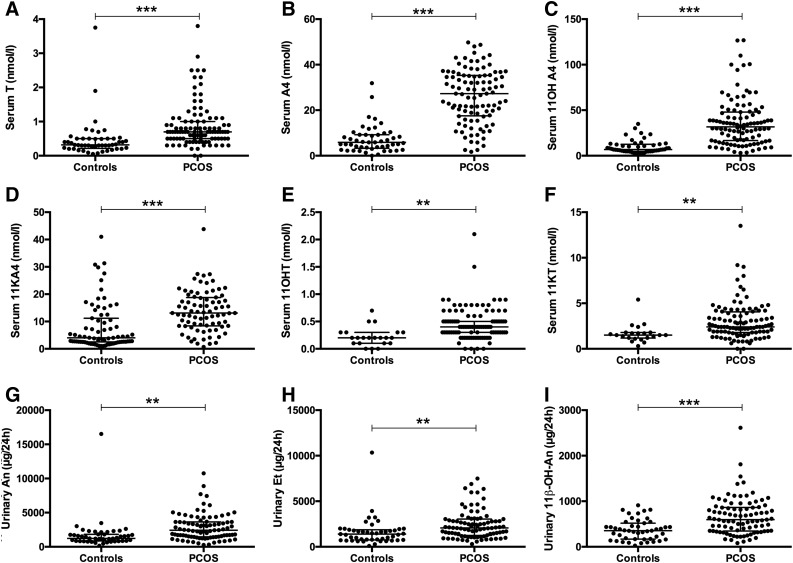
Serum concentrations of (A, B) classic and (C–F) 11-oxygenated steroids in women with PCOS (n = 114) and healthy sex- and age-matched control subjects (n = 49). (G–I) Major urinary androgen metabolite deriving from the classic androgen pathway, An and etiocholanolone (Et), and the 11*β*-hydroxyandrostenedione metabolite 11*β*-OH-An.

LC-MS/MS analysis of serum androgens in the patients with PCOS revealed significantly increased concentrations of the classic androgen T and its precursors A4 and DHEA (all *P* < 0.001) as well as DHEAS (*P* = 0.002) when compared with control subjects (Fig. 2).

**Figure 3.**
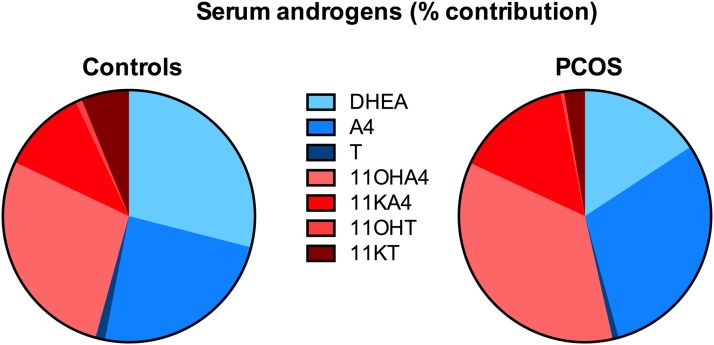
Relative contribution (median; %) of the classic androgen pathway (DHEA, A4, T; shades of blue) and the 11-oxygenated C19 steroid pathway (11OHA4, 11KA4, 11OHT, 11KT; shades of red) to the total circulating androgenic steroid pool, comparing patients with PCOS (n = 114) with sex- and age-matched healthy control subjects (n = 49).

Serum concentrations of the 11-oxygenated androgens 11OHA4, 11OHT, 11KA4, and 11KT were all significantly higher in the PCOS cohort (*P* < 0.001 for 11OHA4 and 11KA4; *P* < 0.01 for 11OHT and 11KT) (Fig. 2). Indeed, the proportionate contribution of 11-oxygenated steroids to total serum androgens was significantly higher in patients with PCOS than in control subjects [53.0% (IQR, 48.7 to 60.3) *vs* 44.0% (IQR, 32.9 to 54.9); *P* < 0.0001] (Fig. 3). Serum concentrations of 11KT were significantly higher than those of T in control subjects and women with PCOS (*P* < 0.001 for both). It should be noted that the lower limit of quantification for 11OHT was 0.65 nmol/L; although 11OHT was detected in the majority of samples, only a single control patient had a value above the lower limit of quantification. By contrast, 22 patients with PCOS had values above this cut-off.

Correspondingly, urinary 24-hour excretion of the classic androgen metabolites An and Et was significantly higher in women with PCOS (*P* = 0.004 and 0.005, respectively) (Fig. 2).

Similarly, the excretion of the urinary metabolite of 11OHA4, 11*β*-OH-An, was significantly increased in patients with PCOS [595 (IQR, 347 to 861) *vs* 353 (IQR, 171 to 487) in control subjects; *P* < 0.001) (Fig. 2).

### 11-oxygenated androgens are increased in PCOS independent of BMI

Serum concentrations of classic androgens (T, A4, DHEA, and DHEAS) and 11-oxygenated androgens (11OHA4, 11KA4, 11OHT, and 11KT) did not differ significantly between obese and nonobese women with PCOS. Similarly, urinary excretion of classic androgen metabolites An, Et, and DHEA and the 11-oxygenated metabolite 11*β*-OH-An were similar in nonobese and obese women with PCOS.

We examined this further by comparing the nonobese PCOS group (n = 51) with healthy control subjects (n = 49). Nonobese women with PCOS were matched for age, BMI, and HOMA-IR with healthy control subjects (Table 1). Serum concentrations of the classic androgens T (*P* < 0.01), A4 (*P* < 0.001), DHEA (*P* < 0.05), and DHEAS (*P* < 0.01) were all higher in the nonobese PCOS cohort than in BMI-matched control subjects. Mirroring this, serum levels of 11OHA4 and 11KA4 (*P* < 0.001 for both) as well as 11OHT (*P* < 0.01) were all significantly increased in nonobese women with PCOS compared with control subjects. There was a trend toward higher serum levels of 11KT in the nonobese PCOS group, but this did not reach statistical significance (*P* = 0.10).

Looking at urinary androgen metabolism, excretion of the 11-oxygenated androgen metabolite 11*β*-OH-An was significantly higher in the nonobese PCOS cohort [595 (IQR, 438 to 841) *vs* 353 (IQR, 171 to 487) in controls; *P* = 0.002], whereas there was only a trend toward significantly higher urinary excretion of An (*P* = 0.07). Excretion of Et and DHEA did not differ between nonobese women with PCOS and control subjects. Serum 11OHA4, 11KA4, 11OHT, and 11KT all correlated significantly with urinary excretion of 11*β*-OH-An (*R* = 0.39, 0.39, 0.33, and 0.40, respectively; *P* < 0.01 for each; for details see Supplemental Table 2).

### 11-oxygenated androgens correlate with markers of metabolic risk

The 11-oxygenated androgen precursors 11OHA4 and 11KA4 correlated significantly with BMI (*R* = 0.31, *P* < 0.01 and *R* = 0.37, *P* < 0.01, respectively). Both also had significant correlations with insulin (*R* = 0.19, *P* < 0.05 and *R* = 0.30, *P* < 0.01, respectively) and HOMA-IR (*R* = 0.21, *P* < 0.05 and *R* = 0.32, *P* < 0.01, respectively). By contrast, we did not observe significant associations with BMI, insulin, or HOMA-IR for 11KT and 11OHT.

The free androgen index (FAI) (serumTx100/SHBG) correlated significantly with BMI, insulin, and HOMA-IR (*P* < 0.01 for each) (Table 2). The FAI also correlated positively with all serum 11-oxygenated androgens. Similar to the findings with 11OHA4 and 11KA4, serum A4 correlated with BMI, insulin, and HOMA-IR (*R* = 0.44, 0.27, and 0.29, respectively; all *P* < 0.01). Serum T correlated weakly with BMI only (*R* = 0.17, *P* < 0.05), whereas serum DHEA and DHEAS did not correlate with BMI, insulin, or HOMA-IR.

**Table 2.** **Correlation Analysis (Spearman’s Rho, All Patients) for Serum Androgens with Baseline Demographics and Metabolic Data in the PCOS and Control Cohorts (n = 163)**

	**Age**	**BMI**	**Glucose**	**Insulin**	**HOMA-IR**	**T**	**A4**	**DHEA**	**DHEAS**	**11OHA4**	**11KA4**	**11OHT**	**11KT**	**FAI**
Age		0.15	0.188*^a^*	−0.08	−0.042	−0.119	0.082	−0.166	−0.255*^b^*	−0.038	−0.017	−0.004	−0.005	−0.108
BMI	0.15		0.330*^b^*	0.587*^b^*	0.602*^b^*	0.170*^a^*	0.439*^b^*	0.112	0.02	0.308*^b^*	0.371*^b^*	0.121	0.171	0.464*^b^*
Glucose	0.188*^a^*	0.330*^b^*		0.332*^b^*	0.440*^b^*	−0.074	−0.004	−0.05	−0.021	0.054	0.011	0.069	0.037	0.075
Insulin	−0.08	0.587*^b^*	0.332*^b^*		0.994*^b^*	0.137	0.274*^b^*	0.129	0.054	0.198*^a^*	0.302*^b^*	−0.033	0.077	0.396*^b^*
HOMA-IR	−0.042	0.602*^b^*	0.440*^b^*	0.994*^b^*		0.112	0.287*^b^*	0.118	0.043	0.215*^a^*	0.316*^b^*	−0.024	0.065	0.380*^b^*
T	−0.119	0.170*^a^*	−0.074	0.137	0.112		0.352*^b^*	0.335*^b^*	0.390*^b^*	0.379*^b^*	0.420*^b^*	0.359*^b^*	0.185*^a^*	0.814*^c^*
A4	0.082	0.439*^b^*	−0.004	0.274*^b^*	0.287*^b^*	0.352*^b^*		0.589*^b^*	0.237*^b^*	0.818*^b^*	0.864*^b^*	0.267*^b^*	0.519*^b^*	0.515*^b^*
DHEA	−0.166*^a^*	0.112	−0.05	0.129	0.118	0.335*^b^*	0.589*^b^*		0.489*^b^*	0.620*^b^*	0.580*^b^*	0.512*^b^*	0.537*^b^*	0.438*^b^*
DHEAS	−0.255*^b^*	0.02	−0.021	0.054	0.043	0.390*^b^*	0.237*^b^*	0.489*^b^*		0.351*^b^*	0.349*^b^*	0.437*^b^*	0.289*^b^*	0.433*^b^*
11OHA4	−0.038	0.308*^b^*	0.054	0.198*^a^*	0.215*^a^*	0.379*^b^*	0.818*^b^*	0.620*^b^*	0.351*^b^*		0.883*^b^*	0.461*^b^*	0.514*^b^*	0.475*^b^*
11KA4	−0.017	0.371*^b^*	0.011	0.302*^b^*	0.316*^b^*	0.420*^b^*	0.864*^b^*	0.580*^b^*	0.349*^b^*	0.883*^b^*		0.373*^b^*	0.595*^b^*	0.526*^b^*
11OHT	−0.004	0.121	0.069	−0.033	−0.024	0.359*^b^*	0.267*^b^*	0.512*^b^*	0.437*^b^*	0.461*^b^*	0.373*^b^*		0.516*^b^*	0.346*^b^*
11KT	−0.005	0.171	0.037	0.077	0.065	0.185*^a^*	0.519*^b^*	0.537*^b^*	0.289*^b^*	0.514*^b^*	0.595*^b^*	0.516*^b^*		0.23*^a^*
FAI	−0.108	0.464*^b^*	0.075	0.396*^b^*	0.380*^b^*	0.814*^c^*	0.515*^b^*	0.438*^b^*	0.433*^b^*	0.475*^b^*	0.526*^b^*	0.346*^b^*	0.23*^a^*	

^*a*^ Significant at *P* < 0.05.

^*b*^ Significant at *P* < 0.01.

^*c*^ Significant at *P* < 0.001.

## Discussion

We have performed a comprehensive comparison of classic and 11-oxygenated androgens in women with PCOS. We found that all measured 11-oxygenated steroids, including the potent androgen 11KT, were significantly increased in women with PCOS compared with control subjects. Previous studies from the early 1990s had reported increased levels of 11OHA4 in PCOS. However, those studies were mostly reliant on immunoassays rather than sensitive and specific mass spectrometry and contained no data on further downstream metabolism of 11OHA4 (12–14). Intriguingly, we could show that 11-oxygenated androgens constitute the majority of the circulating, unconjugated androgen excess in PCOS, which strongly suggests that they are important contributors to PCOS-related hyperandrogenism. Although it could be argued that the shift in favor of 11-oxygenated androgens is due to the abundance of the inactive androgen precursors 11OHA4 (34.9%) and 11KA4 (16.4%), the levels of active 11-oxygenated androgens also exceeded those of classical androgens. Indeed, median circulating concentrations of 11KT were more than threefold higher than those of T in PCOS, traditionally the androgen measured most commonly in clinical practice in the disorder. It is therefore highly likely that 11KT is a major player in PCOS-related androgen excess, especially given that 11KT can activate the androgen receptor in a similar manner to T (9–11, 19–21), and can be converted to the even more potent androgen 11-ketodihydrotestosterone in peripheral target tissue (10, 11). Further studies are required to delineate the relative contributions of these androgens and their downstream activation in target tissues on metabolic risk in PCOS. These data also underpin the crucial role of the adrenal gland in contributing to PCOS-related androgen excess because the first step of the 11-oxygenated androgen pathway is dependent on the 11*β*-hydroxylation of A4 to 11OHA4 by adrenal cytochrome P450 11*β*-hydroxylase.

These results are in broad agreement with a recent study that investigated the contribution of 11-oxygenated androgens to the androgen pool in another androgen excess state, classic congenital adrenal hyperplasia due to 21-hydroxylase deficiency (21OHD). In their study, Turcu *et al.* (22) found that 11OHA4, 11OHT, 11KA4, and 11KT were all significantly elevated in male and female patients with 21OHD when compared with age- and sex-matched control subjects. The authors concluded that the 11-oxygenated androgens represent potentially novel biomarkers of adrenal-derived androgen excess and that 11KT may be the most clinically relevant androgen in patients with 21OHD. However, there are differences in the absolute serum concentrations of the 11-oxygenated steroids reported for the control samples measured in our study and that of Turcu *et al.* (22). Rege *et al.* (9) have also previously measured the levels of 11-oxygenated C19 steroids in the adrenal vein and peripheral circulation of female patients with primary aldosteronism. For comparison, we have pulled together the data from the 2 previous studies in direct comparison with our results in Table 3. In all cases, 11OHA4 is the most abundant 11-oxygenated C19 steroid, and 11KT levels are higher than those of 11OHT. Differences in absolute levels may be ascribed to differences in the composition of the control groups, sample sizes, and sex-specific variability as well as differences in extraction and LC-MS/MS protocols (23). For example, the control group for the 21OHD study consisted of both male (n = 19) and female (n = 19) subjects, with an age range between 3 and 59 years, whereas the control group from this study consisted only of female subjects (n = 49) aged 18 to 40 years. To the best of our knowledge, deuterated internal standards for 11KA4, 11OHT, and 11KT are not currently commercially available, thereby complicating the quantification of these steroids by LC-MS/MS. Nonetheless, comparisons between control groups and patient groups within these individual studies unequivocally demonstrate that the 11-oxygenated androgens are significantly elevated in both PCOS and 21OHD. Further studies with larger cohorts, as well as with the use of deuterated 11-oxygenated internal standards, are therefore required to establish reliable reference ranges in health and disease.

**Table 3.** **Serum Concentrations of Classical and 11-Oxygenated Androgens**

**Steroid (nmol/L)**	**Adrenal Vein Study—Rege *et al.* (****9****), Mean ± SEM**		**CAH Study—Turcu *et al.* (****22****), Median (IQR)**		**PCOS—This Study, Median (IQR)**	
	**Adrenal Vein (n = 7; all women)**	**Periphery (n = 7; all women)**	**Controls (n = 38; 19 women)**	**21OHD (n = 38; 19 women)**	**Controls (n = 49; all women)**	**PCOS (n = 114; all women)**
DHEAS	3827 ± 1317	2210 ± 321	3793.4 (1585.1–5066.5)	508.7 (213.0–1745.2)	6038 (3402–9522)	8133 (5515–12,240)
DHEA	125 ± 56.9	5.85 ± 1.01	6.0 (4.1–11.0)	1.0 (0.55–2.9)	7.1 (4.2–11.8)	14.1 (10.4–18.2)
A4	79.0 ± 46.9	1.90 ± 0.47	1.5 (0.77–2.2)	5.4 (2.5–13.6)	5.9 (3.6–9.2)	26.8 (16.9–35.2)
T	0.78 ± 0.26	0.44 ± 0.05	0.90 (0.42–10.7)	2.8 (1.3–5.6)	0.3 (0.2–0.5)	0.7 (0.5–1.0)
11OHA4	157 ± 96.2	1.90 ± 0.42	3.9 (2.3–5.1)	11.6 (6.2–26.2)	6.8 (4.9–12.3)	31.7 (16.8–47.8)
11KA4	0.99 ± 0.33	0.46 ± 0.07	1.0 (0.67–1.4)	3.2 (1.9–4.8)	2.7 (2.0–3.8)	13.4 (8.5–18.8)
11OHT	0.48 ± 0.17	0.22 ± 0.04	0.49 (0.30–0.69)	1.9 (0.69–3.4)	0.2 (0.1–0.3)	0.4 (0.3–0.5)
11KT	0.39 ± 0.09	0.44 ± 0.03	1.7 (0.96–2.6)	5.7 (3.5–12.1)	1.5 (1.2–1.8)	2.4 (1.8–3.9)

Data are as measured in 3 separate studies: a comparison of adrenal vein and peripheral blood concentrations in women with primary aldosteronism (9); healthy control subjects *vs* patients with congenital adrenal hyperplasia due to 21-hydroxylase deficiency receiving routine steroid therapy (22); and this study comparing PCOS with healthy control subjects.

Abbreviations: CAH, congenital adrenal hyperplasia; SEM, standard error of the mean.

This study has validated our observation from previously published work that A4 is a surrogate marker of metabolic risk in PCOS, confirming significant positive associations with BMI, fasting insulin, and HOMA-IR (5). In this study we found that the serum concentrations of the 11-oxygenated androgen precursors 11OHA4 and 11KA4 also significantly correlate with BMI, fasting insulin, and HOMA-IR. Hence, these steroids may have additional value as surrogate markers of metabolic risk in PCOS. This finding is perhaps not entirely surprising given that 11OHA4 and 11KA4 are derivatives of A4 and that highly significant correlations are observed between all 3 steroids (Table 2).

Not only obese but also nonobese women with PCOS had significantly higher levels of 11OHA4, 11KA4, and 11OHT than BMI-matched healthy control subjects, and levels of all 11-oxygenated androgens did not differ between obese and nonobese women with PCOS. By contrast, HOMA-IR was only significantly increased in the obese patients. It is unclear why the serum levels of 11OHA4 and 11KA4 did not differ between obese and nonobese women with PCOS given the above-described significant association of these steroids with BMI. Unfortunately, our patient number did not allow a correlation analysis split by subgroup with sufficient statistical power. A4 is already a suitable surrogate marker of metabolic risk in PCOS. It is, however, important that future studies investigate the role of these androgen precursors in metabolic target tissues because their metabolism and consequent activity may well differ.

Our results may also suggest that androgen excess precedes androgen-driven insulin resistance and subsequent weight gain in the pathophysiological sequence of PCOS. Androgen-mediated effects on adipose tissue function and fat mass expansion are increasingly recognized (24), and classic androgen pathway serum androgens correlate closely with adipose tissue mass in women (25). Prenatal androgen exposure is associated with adipocyte hypertrophy and increased fat mass in rodent and sheep models (26, 27). Women with early-onset androgen excess in PCOS may therefore be predisposed to androgen-mediated obesity in later adulthood, fueling a vicious circle of androgen excess, weight gain, and hyperinsulinemia (5).

A key factor in peripheral androgen metabolism is aldo-keto reductase type 1C3 (AKR1C3), which converts A4 to T. AKR1C3 is highly expressed in adipose tissue (28), and its expression in subcutaneous adipose tissue is increased not only in subjects with simple obesity (29) but also in women with PCOS (30). In our study, BMI correlated significantly with serum T, supporting the conversion of A4 to T by AKR1C3 in adipose tissue. Conversely, 11OHA4 is not a substrate for AKR1C3 (10) and thus cannot be converted to 11OHT in adipose tissue. Similarly, although 11KA4 is a substrate for AKR1C3 (10), the expression of 11*β*-hydroxysteroid dehydrogenase type 1 in adipose tissue (31) may minimize its conversion to 11KT because 11*β*HSD1 efficiently converts 11KA4 to 11OHA4, which is not a substrate for AKR1C3 (10, 32). These conversions are supported by the observation that although 11OHT and 11KT levels are significantly elevated in PCOS, neither 11OHT nor 11KT correlated with BMI. This finding further supports the idea that androgen excess drives weight gain in PCOS. Our observations therefore provide further evidence for a causal link between androgen excess and metabolic dysfunction in PCOS.

In summary, we have demonstrated that 11-oxygenated androgens are significantly elevated in obese and nonobese women with PCOS and cumulatively constitute a greater proportion of total circulating androgens than classic androgens. This observation has not been replicated in healthy control subjects, where classic androgens appear to constitute the majority of the circulating androgen pool. Intriguingly, 11KT circulates in significantly higher concentrations than T, both in women with PCOS and control subjects, opening up avenues for the exploring the origins of androgen excess in PCOS. Close correlation of a number of 11-oxygenated steroids with hyperinsulinemia further highlight a potential important role for these steroids as biomarkers not only of androgen excess but also of insulin resistance, metabolic dysfunction, and tissue-specific androgen activation in PCOS.

## References

[1] Rotterdam ESHRE/ASRM-Sponsored PCOS consensus workshop group Revised 2003 consensus on diagnostic criteria and long-term health risks related to polycystic ovary syndrome (PCOS). Hum Reprod. 2004;19(1):41–47. 14688154 10.1093/humrep/deh098

[2] Stewart PM, Shackleton CH, Beastall GH, Edwards CR 5 alpha-reductase activity in polycystic ovary syndrome. Lancet. 1990;335(8687):431–433. 1968168 10.1016/0140-6736(90)90664-q

[3] Vassiliadi DA, Barber TM, Hughes BA, McCarthy MI, Wass JA, Franks S, Nightingale P, Tomlinson JW, Arlt W, Stewart PM Increased 5 alpha-reductase activity and adrenocortical drive in women with polycystic ovary syndrome. J Clin Endocrinol Metab. 2009;94(9):3558–3566. 19567518 10.1210/jc.2009-0837

[4] Torchen LC, Idkowiak J, Fogel NR, O’Neil DM, Shackleton CH, Arlt W, Dunaif A Evidence for increased 5α-reductase activity during early childhood in daughters of women with polycystic ovary syndrome. J Clin Endocrinol Metab. 2016;101(5):2069–2075. 26990942 10.1210/jc.2015-3926 PMC4870855

[5] O’Reilly MW, Taylor AE, Crabtree NJ, Hughes BA, Capper F, Crowley RK, Stewart PM, Tomlinson JW, Arlt W Hyperandrogenemia predicts metabolic phenotype in polycystic ovary syndrome: the utility of serum androstenedione. J Clin Endocrinol Metab. 2014;99(3):1027–1036. 24423344 10.1210/jc.2013-3399 PMC3955250

[6] Pasquali R, Zanotti L, Fanelli F, Mezzullo M, Fazzini A, Morselli Labate AM, Repaci A, Ribichini D, Gambineri A Defining hyperandrogenism in women with polycystic ovary syndrome: a challenging perspective. J Clin Endocrinol Metab. 2016;101(5):2013–2022. 26964728 10.1210/jc.2015-4009

[7] Conway G, Dewailly D, Diamanti-Kandarakis E, Escobar-Morreale HF, Franks S, Gambineri A, Kelestimur F, Macut D, Micic D, Pasquali R, Pfeifer M, Pignatelli D, Pugeat M, Yildiz BO; ESE PCOS Special Interest Group The polycystic ovary syndrome: a position statement from the European Society of Endocrinology. Eur J Endocrinol. 2014;171(4):P1–P29. 24849517 10.1530/EJE-14-0253

[8] Pretorius E, Arlt W, Storbeck KH A new dawn for androgens: novel lessons from 11-oxygenated C19 steroids [published online ahead of print August 9, 2016]. Mol Cell Endocrinol. 10.1016/j.mce.2016.08.014 27519632

[9] Rege J, Nakamura Y, Satoh F, Morimoto R, Kennedy MR, Layman LC, Honma S, Sasano H, Rainey WE Liquid chromatography-tandem mass spectrometry analysis of human adrenal vein 19-carbon steroids before and after ACTH stimulation. J Clin Endocrinol Metab. 2013;98(3):1182–1188. 23386646 10.1210/jc.2012-2912 PMC3590473

[10] Storbeck KH, Bloem LM, Africander D, Schloms L, Swart P, Swart AC 11β-Hydroxydihydrotestosterone and 11-ketodihydrotestosterone, novel C19 steroids with androgenic activity: a putative role in castration resistant prostate cancer? Mol Cell Endocrinol. 2013;377(1-2):135–146. 23856005 10.1016/j.mce.2013.07.006

[11] Pretorius E, Africander DJ, Vlok M, Perkins MS, Quanson J, Storbeck KH 11-ketotestosterone and 11-ketodihydrotestosterone in castration resistant prostate cancer: potent androgens which can no longer be ignored. PLoS One. 2016;11(7):e0159867. 27442248 10.1371/journal.pone.0159867 PMC4956299

[12] Carmina E, Stanczyk FZ, Chang L, Miles RA, Lobo RA The ratio of androstenedione:11 beta-hydroxyandrostenedione is an important marker of adrenal androgen excess in women. Fertil Steril. 1992;58(1):148–152. 1623996 10.1016/s0015-0282(16)55152-8

[13] Holownia P, Owen EJ, Conway GS, Round J, Honour JW Studies to confirm the source of 11 beta-hydroxyandrostenedione. J Steroid Biochem Mol Biol. 1992;41(3-8):875–880. 1532906 10.1016/0960-0760(92)90441-k

[14] Owen EJ, Holownia P, Conway GS, Jacobs HS, Honour JW 11 beta-hydroxyandrostenedione in plasma, follicular fluid, and granulosa cells of women with normal and polycystic ovaries. Fertil Steril. 1992;58(4):713–718. 1426315

[15] Chadwick CA, Owen LJ, Keevil BG Development of a method for the measurement of dehydroepiandrosterone sulphate by liquid chromatography-tandem mass spectrometry. Ann Clin Biochem. 2005;42(6):468–474. 16259799 10.1258/000456305774538175

[16] Quanson JL, Stander MA, Pretorius E, Jenkinson C, Taylor AE, Storbeck KH High-throughput analysis of 19 endogenous androgenic steroids by ultra-performance convergence chromatography tandem mass spectrometry. J Chromatogr B Analyt Technol Biomed Life Sci. 2016;1031:131–138. 10.1016/j.jchromb.2016.07.024 27479683

[17] Büttler RM, Martens F, Fanelli F, Pham HT, Kushnir MM, Janssen MJ, Owen L, Taylor AE, Soeborg T, Blankenstein MA, Heijboer AC Comparison of 7 published LC-MS/MS methods for the simultaneous measurement of testosterone, androstenedione, and dehydroepiandrosterone in serum. Clin Chem. 2015;61(12):1475–1483. 26503965 10.1373/clinchem.2015.242859

[18] Arlt W, Walker EA, Draper N, Ivison HE, Ride JP, Hammer F, Chalder SM, Borucka-Mankiewicz M, Hauffa BP, Malunowicz EM, Stewart PM, Shackleton CH Congenital adrenal hyperplasia caused by mutant P450 oxidoreductase and human androgen synthesis: analytical study. Lancet. 2004;363(9427):2128–2135. 15220035 10.1016/S0140-6736(04)16503-3

[19] Yazawa T, Uesaka M, Inaoka Y, Mizutani T, Sekiguchi T, Kajitani T, Kitano T, Umezawa A, Miyamoto K Cyp11b1 is induced in the murine gonad by luteinizing hormone/human chorionic gonadotropin and involved in the production of 11-ketotestosterone, a major fish androgen: conservation and evolution of the androgen metabolic pathway. Endocrinology. 2008;149(4):1786–1792. 18162527 10.1210/en.2007-1015

[20] Campana C, Rege J, Turcu AF, Pezzi V, Gomez-Sanchez CE, Robins DM, Rainey WE Development of a novel cell based androgen screening model. J Steroid Biochem Mol Biol. 2016;156:17–22. 26581480 10.1016/j.jsbmb.2015.11.005 PMC4748855

[21] Imamichi Y, Yuhki KI, Orisaka M, Kitano T, Mukai K, Ushikubi F, Taniguchi T, Umezawa A, Miyamoto K, Yazawa T 11-Ketotestosterone is a major androgen produced in human gonads. J Clin Endocrinol Metab. 2016;101(10):3582–3591. 27428878 10.1210/jc.2016-2311

[22] Turcu AF, Nanba AT, Chomic R, Upadhyay SK, Giordano TJ, Shields JJ, Merke DP, Rainey WE, Auchus RJ Adrenal-derived 11-oxygenated 19-carbon steroids are the dominant androgens in classic 21-hydroxylase deficiency. Eur J Endocrinol. 2016;174(5):601–609. 26865584 10.1530/EJE-15-1181 PMC4874183

[23] Keevil BG LC-MS/MS analysis of steroids in the clinical laboratory. Clin Biochem. 2016;49(13-14):989–997. 27131495 10.1016/j.clinbiochem.2016.04.009

[24] O’Reilly MW, House PJ, Tomlinson JW Understanding androgen action in adipose tissue. J Steroid Biochem Mol Biol. 2014;143:277–284. 24787657 10.1016/j.jsbmb.2014.04.008

[25] Mongraw-Chaffin ML, Anderson CA, Allison MA, Ouyang P, Szklo M, Vaidya D, Woodward M, Golden SH Association between sex hormones and adiposity: qualitative differences in women and men in the multi-ethnic study of atherosclerosis. J Clin Endocrinol Metab. 2015;100(4):E596–E600. 25636047 10.1210/jc.2014-2934 PMC4399289

[26] Yan X, Dai X, Wang J, Zhao N, Cui Y, Liu J Prenatal androgen excess programs metabolic derangements in pubertal female rats. J Endocrinol. 2013;217(1):119–129. 23426873 10.1530/JOE-12-0577

[27] Nada SE, Thompson RC, Padmanabhan V Developmental programming: differential effects of prenatal testosterone excess on insulin target tissues. Endocrinology. 2010;151(11):5165–5173. 20843997 10.1210/en.2010-0666 PMC2954716

[28] Blouin K, Blanchette S, Richard C, Dupont P, Luu-The V, Tchernof A Expression and activity of steroid aldoketoreductases 1C in omental adipose tissue are positive correlates of adiposity in women. Am J Physiol Endocrinol Metab. 2005;288(2):E398–E404. 15494612 10.1152/ajpendo.00312.2004

[29] Quinkler M, Sinha B, Tomlinson JW, Bujalska IJ, Stewart PM, Arlt W Androgen generation in adipose tissue in women with simple obesity--a site-specific role for 17beta-hydroxysteroid dehydrogenase type 5. J Endocrinol. 2004;183(2):331–342. 15531721 10.1677/joe.1.05762

[30] Wang L, Li S, Zhao A, Tao T, Mao X, Zhang P, Liu W The expression of sex steroid synthesis and inactivation enzymes in subcutaneous adipose tissue of PCOS patients. J Steroid Biochem Mol Biol. 2012;132(1-2):120–126. 22381227 10.1016/j.jsbmb.2012.02.003

[31] Tomlinson JW, Moore JS, Clark PM, Holder G, Shakespeare L, Stewart PM Weight loss increases 11beta-hydroxysteroid dehydrogenase type 1 expression in human adipose tissue. J Clin Endocrinol Metab. 2004;89(6):2711–2716. 15181046 10.1210/jc.2003-031376 PMC7611657

[32] Swart AC, Schloms L, Storbeck KH, Bloem LM, Toit Td, Quanson JL, Rainey WE, Swart P 11β-hydroxyandrostenedione, the product of androstenedione metabolism in the adrenal, is metabolized in LNCaP cells by 5α-reductase yielding 11β-hydroxy-5α-androstanedione. J Steroid Biochem Mol Biol. 2013;138:132–142. 23685396 10.1016/j.jsbmb.2013.04.010

