# Supplementary material for: 11-Oxygenated C19 Steroids Are the Predominant Androgens in Polycystic Ovary Syndrome
Source: J Clin Endocrinol Metab. 2016 Nov 30;102(3):840–8. doi: 10.1210/jc.2016-3285 (PMC5460696; doi:10.1210/jc.2016-3285)
Supplement: Supplementary file 1 [file jc.2016-3285.st1.pdf]

Supplementary Table 1: Method validation data for the liquid chromatography-tandem mass spectrometry (LC-MS/MS) assay used to profile 11-oxygenated C19 steroids: Lower Limit of Quantification (LLOQ) (ng/mL); r^2; precision (%RSD) and accuracy (%). Validation was performed in accordance to US FDA guidelines*. Abbreviations: 11OHA4, 11β-hydroxyandrostenedione; 11KA4, 11-ketoandrostenedione; 11OHT, 11β-hydroxytestosterone; 11KT, 11-ketotestosterone; LLOQ, lower limit of quantification; RSD, relative standard deviation.

Steroid	LLOQ (nmol/L)	r^2	Concentration (nnol/L)	Level	Precision (%RSD) Intra-day (n=6)	Precision (%RSD) Inter-day (n=6+6+6)	Accuracy (%) (n=6)
			0.66	LLOQ			
11OHA4	0.83	0.9869	1.98	Low	9.5	9.3	99.4
			24.8	Medium	2.4	7.0	98.8
			132.3	High	4.8	7.6	101.5
			0.67	LLOQ	7.0	7.2	117.5
11KA4	0.83	0.9967	1.99	Low	6.7	8.0	105.7
			24.9	Medium	2.6	4.9	101.5
			133.2	High	4.3	4.5	96.7
			0.65	LLOQ	6.6	5.7	108.0
11OHT	0.65	0.9988	1.97	Low	4.8	8.7	104.8
			24.6	Medium	5.4	7.7	103.7
			131.4	High	5.6	5.0	101.2
			0.66	LLOQ	6.2	11.0	113.4
11KT	0.17	0.9985	1.98	Low	4.8	9.4	94.8
			24.8	Medium	8.4	9.1	90.0
			132.3	High	3.8	9.8	102.9

*United States Food and Drug Administration (USFDA). Guidance for Industry Bioanalytical Method Validation. 2001. www.fda.gov/downloads/Drugs/Guidances/ucm070107.pdf.

Supplementary Table 2: Correlation analysis (Spearman's Rho) between the concentrations of the steroids of the classic and the 11-oxygenated androgen synthesis pathways and urinary androgen metabolite excretion in all subjects (n=163).

	Classic androgens				***11-oxygenated androgens***				***Urinary androgen metabolites***			
	**T**	**A4**	**DHEA**	**DHEAS**	**11OHA4**	**11KA4**	**11OHT**	**11KT**	**U-An**	**U-Et**	**U-DHEA**	**U-11βOH-An**
**T**	-	.352**	.335**	.390**	.379**	.420**	.359**	.185*	.244**	.224*	.071	.243**
**A4**	.352**	-	.589**	.237**	.818**	.864**	.267**	.519**	.353**	.311**	.138	.424**
**DHEA**	.335**	.589**	-	.489**	.620**	.580**	.512**	.537**	.321**	.278**	.126	.355**
**DHEAS**	.390**	.237**	.489**	-	.351**	.349**	.437**	.289**	.135	.190*	.11	.054
**11OHA4**	.379**	.818**	.620**	.351**	-	.883**	.461**	.514**	.298**	.266**	.116	.395**
**11KA4**	.420**	.864**	.580**	.349**	.883**	-	.373**	.595**	.376**	.309**	.167	.390**
**11OHT**	.359**	.267**	.512**	.437**	.461**	.373**	-	.516**	.131	.236*	.215*	.333**
**11KT**	.185*	.519**	.537**	.289**	.514**	.595**	.516**	-	.302**	.278**	.215*	.398**
**U-An**	.244**	.353**	.321**	.135	.298**	.376**	.131	.302**	-	.736**	.520**	.772**
**U-Et**	.224*	.311**	.278**	.190*	.266**	.309**	.236*	.278**	.736**	-	.599**	.747**
**U-DHEA**	.071	.138	.126	.11	.116	.167	.215*	.215*	.520**	.599**	-	.512**
**U-11β-OHAn**	.243**	.424**	.355**	.054	.395**	.390**	.333**	.398**	.772**	.747**	.512**	-

Abbreviations: 11OHA4, 11β-hydroxyandrostenedione; 11KA4, 11-ketoandrostenedione; 11OHT, 11β-hydroxytestosterone; 11KT, 11-ketotestosterone; A4, androstenedione; DHEA, dehydroepiandrosterone; DHEAS, dehydroepiandrosterone sulfate; T, testosterone; U-11β-OH-An, urinary 11β-hydroxyandrosterone; U-An, urinary androsterone; U-Et, urinary etiocholanolone; U-DHEA, urinary DHEA. *$p<0.05$; **$p<0.01$; ***$p<0.001$.
